# Perforation of the Ileum Commencing on the Peritoneal Surface

**Published:** 1844-04-20

**Authors:** 

**Affiliations:** Glasgow


### PERFORATION OF THE ILEUM COMMENCING ON THE PERITONEAL SURFACE.

REPORTED BY DR. ADAMS, OF GLASGOW.

William Mitchell, aged 54, tolerably robust, temperate, and enjoying good health up to May 28, 1840. At ten o'clock, A. M., of that day, I was sent for, and found him affected with all the symptoms of strangulated hernia,—his scrotum filled on both sides with intestine, the left protrusion irreducible. The taxis was successful, and the urgent symptoms almost immediately relieved. At my next visit he complained much of pain in the left iliac region, which a few leeches, followed by warm fomentations, abated considerably. I heard no more of him until Saturday, the 13th of June, and at ten, P. M., on that day, I was again summoned. He had enjoyed good health from the time of my last visit, thirteen days before, up to half an hour prior to the present one; however, the tenderness in the left iliac region had never entirely subsided, and was sufficiently acute to prevent his wearing other than his old bag-truss. He had been unable, also, to reduce, as formerly, the entire hernial protrusion, but that circumstance caused him uneasiness, and his bowels were easily enough regulated. About half an hour before my visit, he had, whilst indulging in the company of some friends, been suddenly seized with a violent pain in the left iliac region, accompanied with an extreme sense of weakness, followed by sickness and violent retching. At my visit the pain had become more diffused over the lower part of the abdomen, which was painful to the touch; the inferior portions of the abdominal muscles were so contracted as to feel as hard as a board; the expression of countenance was exceedingly anxious, and the features were greatly altered in appearance; the whole surface of the body was cold, and covered with a profuse perspiration; the pulse was 120, small and thready; the sickness and vomiting still continued. With respect to the herniæ, both protruded considerably, but could be returned with tolerable facility; excepting a doughy mass, which had been felt in the left hernial sac, even after it was emptied of the great portion of its contents. From the large size of the abdominal aperture, from the ease with which the greater portion of the hernia could be reduced, and from the statement of the patient, that it had never been more so since my last visit, I was satisfied that there was no strangulation of intestine to account for the symptoms then present. I accordingly diagnosed peritonitis from perforation of intestine. The remedial measures were turpentine embrocations to the abdomen, and, every third hour, three grains of calomel and one of opium. At ten o'clock next morning the patient's appearance was highly satisfactory. He had slept a little during the night; the expression of his countenance was improved; there was less abdominal tenderness; the surface of the body was warmer; the pulse somewhat fuller and less frequent; his bowels were still, however, unopened, and his efforts at micturition were frequent, unsatisiactory, and accompanied with excessive pain. The calomel and opium, with the turpentine embrocations were continued, and the catheter was used. The appearances of amendment in the patient's condition proved fallacious. At two in the afternoon, when I again saw him he was evidently moribund. The efforts at vomiting had returned shortly afier the last visit, and still continued; the pulse was imperceptible at the wrist; cold sweats again bedewed the body; the countenance was anxious in the extreme; the breathing panting, and accompanied with the tracheal rattle. The patient, who was quite sensible, said he had no pain. He died at six o'clock the same evening. Thus, the time which elapsed beetween the last sudden attack and its fatal termination was exactly twenty and a half hours.

### *Examination Thirty-eight hours after Death.*

The body was plump, on many places showed livid patches, and exhaled a most offensive odour; the abdomen was very tympanitic, and the scrotum inflated to an enormous extent; on laying open the abdomen, fetid air gushed out in great abundance; the large omentum was much upon the stretch, and at one point adhered strongly to the left hernial sac; on turning it over, the small intestines were seen highly injected, streaked with lymph, and in many places covered with purulent matter, they did not, however, adhere to each other, nor was any portion of them engaged in either hernial sac. In the pelvis and the right and left lumbar regions, we found a considerable quantity of a dark-brown colored fluid, having a most offensive feculent smell; and on examining more minutely the state of the intestines, we found, in addition to the general effects of acute inflammation already described, towards the left side, and in that portion of the intestine usually considered as the commencement of the ileum, a circular perforation, large enough to admit the point of the forefinger. The appearances of the inflammation were more vivid in this situation, and the quantity of lymph and purulent matter thrown out was greater than in the other parts. It was evident that the intestinal tunics were not all destroyed in an equal manner, *the destruction of the peritoneal being considerably greater than that of the mucous.* On the same portion of intestine, and within an inch and a half of the perforation, a small spot of ulceration was discovered, *confined entirely to the serous membrane.* The mucous coat was throughout healthy, and even in the immediate vicinity of the perforation, showed no traces of increased vascular action. We examined most carefully the whole intestinal tract, from the stomach to the rectum, but could discover no further lesion. The mesenteric glands were of their normal volume. The case I consider to be almost unique.—*Edin. Monthly Journal.*

